# Functional optimization of electric cell-substrate impedance sensing (ECIS) using human corneal epithelial cells

**DOI:** 10.1038/s41598-022-18182-z

**Published:** 2022-08-19

**Authors:** Abdul Shukkur Ebrahim, Thanzeela Ebrahim, Hussein Kani, Ahmed S. Ibrahim, Thomas W. Carion, Elizabeth A. Berger

**Affiliations:** 1 Department of Ophthalmology, Visual & Anatomical Sciences, Wayne State University School of Medicine, 540 E. Canfield Avenue, Detroit, MI 48201 USA; 2 Department of Health Sciences, University of Central Florida College of Health Professions and Sciences, Orlando, FL 32816 USA; 3 Department of Pharmacology, Wayne State University School of Medicine, Detroit, MI 48201 USA; 4 Department of Biochemistry, Faculty of Pharmacy, Mansoura University, Mansoura, 35516 Egypt

**Keywords:** Experimental models of disease, Cell migration, Cells

## Abstract

An intact epithelium is key to maintaining corneal integrity and barrier function which can lead to impaired ocular defense and sight-threatening opacity when compromised. Electrical cell-substrate impedance sensing or ECIS is a non-invasive method to measure real-time cellular behaviors including barrier function and cell migration. The current study uses ECIS technology to assess and optimize human telomerase-immortalized corneal epithelial cells to generate quantifiable measurements that accurately reflect changes in cell behavior in vitro. Five cell densities were assessed in two different media to determine the optimal conditions for monitoring of cellular behavior over time. Parameters of evaluation included: overall impedance (Z), barrier resistance (R), cell capacitance (C), and mathematical modeling of the R data to further generate R_b_ (the electrical resistance between HUCLs), α (the resistance between the HUCLs and the substrate), and C_m_ (the capacitance of the cell membrane) measurements. All parameters of assessment strongly indicated DMEM/F12 at 60,000 cells as the optimal condition for ECIS assessment of HUCLs. Furthermore, this work highlights the ability of the sensitive ECIS biosensor technology to comprehensively and quantitatively assess corneal epithelial cell structure and function and the importance of optimizing not only cell density, but choice of media used for in vitro culturing.

## Introduction

Corneal epithelial cells are widely used for various in vitro assessments, including drug toxicity, host–pathogen interactions, and wound healing. Traditionally, these approaches are limited to cell viability and proliferation rates, cell migration as detected using Boyden chambers and scratch assays, and various secondary measurements (cytokine release, downstream signaling pathways) to represent functional changes. Further, the aforementioned methods tend to lack real-time assessments of changes in cellular structure and function. To this end, we have established the current protocol that optimizes in vitro conditions of human telomerase-immortalized corneal epithelial cells (HUCLs) for analysis using electric cell-substrate impedance sensing or ECIS. This powerful approach is a non-invasive method used to continuously monitor cell behavior and integrity, while dynamically measuring and modeling parameter changes in cell migration and barrier function.

The ECIS system is able to quantify multiple barrier-related parameters to represent changes in cellular behavior over time. This robust functional assessment is possible due to ECIS being an alternating current (AC)-based biosensor that measures impedance (Z; Ω), which is comprised of resistance (R; Ω) and capacitance (C; farad or F)^1^. The use of a constant AC of 1 μA with a given frequency as a replacement for a direct current (DC) allows for the separation of overall impedance into overall barrier resistance and cell capacitance^2^. Capacitance measures the overall coverage of the well by the cell layer, whereas resistance is indicative of the barrier function of the epithelial cells^2^. Furthermore, due to the multifrequency nature of ECIS, the impedance data can be mathematically modeled to calculate the intercellular resistance (R_b_; Ω-cm^2^), the basolateral adhesion of the cells to the substrate (α; Ω-cm^1/2^), and the capacitance at the cell membrane (C_m_; µF/Ω-cm^2^)^3^. Use of the ECIS system provides a highly sensitive method to effectively monitor epithelial cell barrier function in a continuous manner and generate quantifiable measurements to evaluate changes in cellular behavior. Although ECIS has been previously used to comparatively evaluate human corneal epithelial cells against TEER^4^, measurements were limited to resistance only; whereas the current work optimizes culture conditions while providing a broader assessment.

The outermost cornea is a self-renewing, layered epithelial sheet that serves as the primary line of defense against noxious stimuli and invading pathogens. If the epithelial barrier is compromised or suboptimal conditions are present within the corneal microenvironment, pathogenic conditions can develop leading to impaired wound healing and progressive visual opacity^5^. In vitro representation of an intact epithelium is integral to studying corneal homeostasis and pathogenic events associated with disease. To this end, human corneal epithelial cell lines are often used and are traditionally grown in keratinocyte serum-free medium (K-SFM) as a standard^6^. These cells effectively represent the apical layer of nonkeratinized squamous cells that form tight junctions between adjacent cells and regulate the passage of molecules, toxins, and fluids in the cell environment^7^. Therefore, the current study sought to optimize growth conditions as assessed by ECIS biosensor technology using HUCLs at different cell densities and culture media. Quantifiable measurements were generated that accurately reflect changes in cell behavior under in vitro conditions. The ECIS assessments provide critical insights into: (1) how long it takes for the epithelial cells to spread and form a confluent monolayer; (2) when the epithelial barrier has formed; (3) when the epithelial barrier is the strongest; (4) contribution of paracellular barrier (R_b_) to overall resistance; and (5) contribution of basal adhesion (α) to overall resistance. Furthermore, we provide clear ECIS-derived evidence that DMEM/F12 media supplemented with 10% FBS is ideal for HUCL growth, attachment, spreading, and barrier formation as opposed to the traditionally used non-supplemented K-SFM media.

## Results

### Three-dimensional bio-impedance analysis

**Figure 1:**
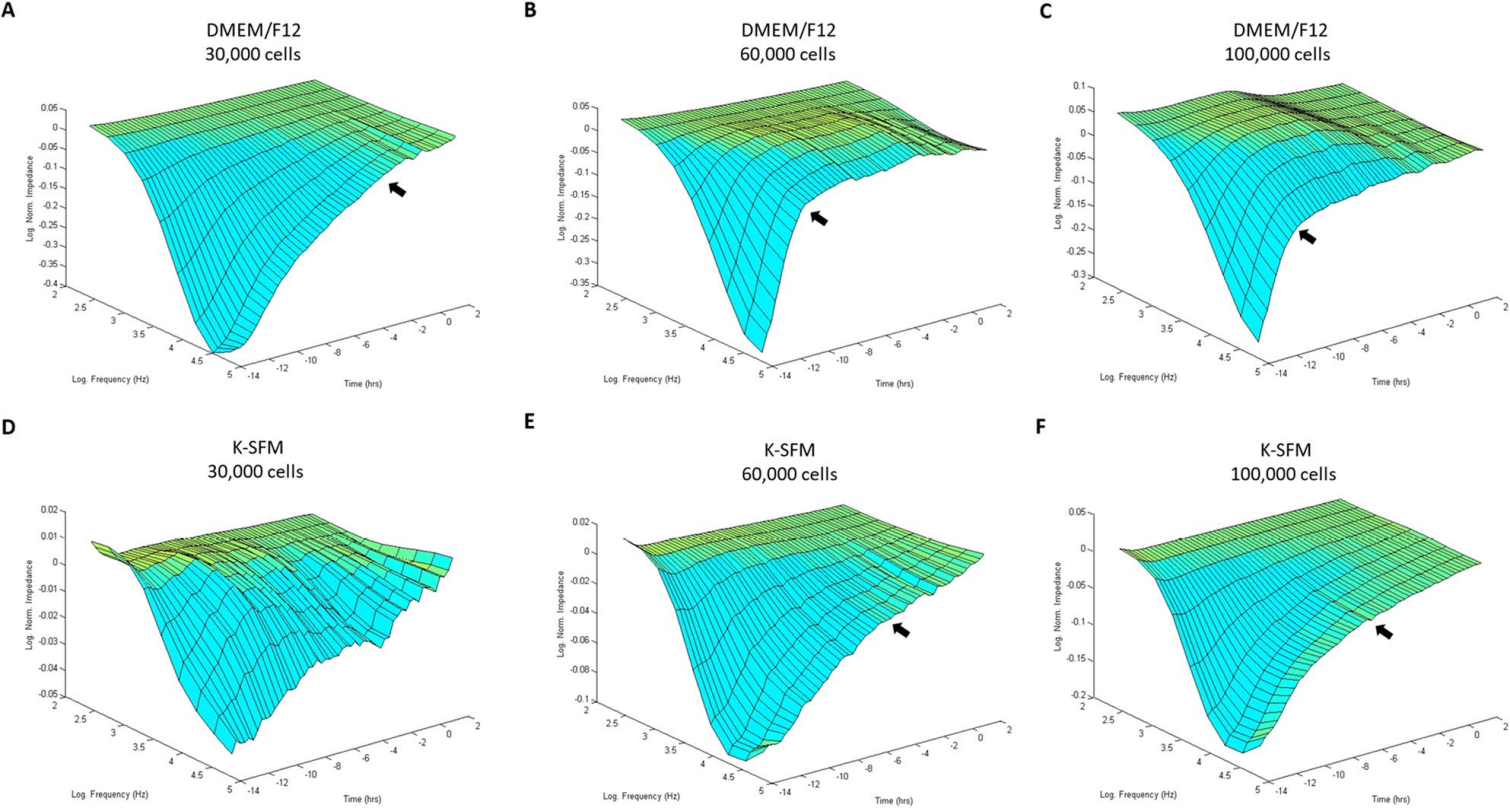
Barrier function of HUCLs monitored by real-time bio impedance analysis using an AC frequency scan. HUCLs were seeded on a 96W1E + ECIS array. Three-dimensional representations of the log of normalized impedance (y-axis) as a function of both log frequency of the alternating-current (AC) (y-axis) and time (z-axis). Cells grown in DMEM/F12 and K-SFM are shown for 30,000 (**A**,**D**), 60,000 (**B**,**E**) and 100,000 (**C**,**F**) cell seeding densities. Arrows indicate start of plateau and approximate time to confluency.

Bio-impedance analysis of HUCLs was carried out to compare two different cell culture media (DMEM/F12 and K-SFM) at three different cell densities (30,000, 60,000 and 100,000 cells per well) as shown in Fig. 1A–F. HUCLs formed a mature confluent barrier as indicated by a plateau in impedance (Z) represented as log normalized values on the y-axis in the 3D model. As such, HUCLs grown in DMEM/F12 media at all three seeding densities (A–C) formed a mature confluent barrier faster than cells similarly grown in K-SFM media (D–F). Furthermore, three-dimensional representations of normalized impedance across HUCLs as a function of both time and log frequency showed DMEM/F12 at a density of 60,000 was most optimal for barrier maturation (B). Likewise, at a 60,000-seeding density, the logarithmic growth curve reached a plateau (time to confluency) after 4–6 h with DMEM/F12 (B) compared to 8–10 h with K-SFM (E). Thus, indicating that HUCLs grown in the supplemented media more efficiently form an epithelial monolayer than cells similarly grown in unsupplemented media.

**Figure 2:**
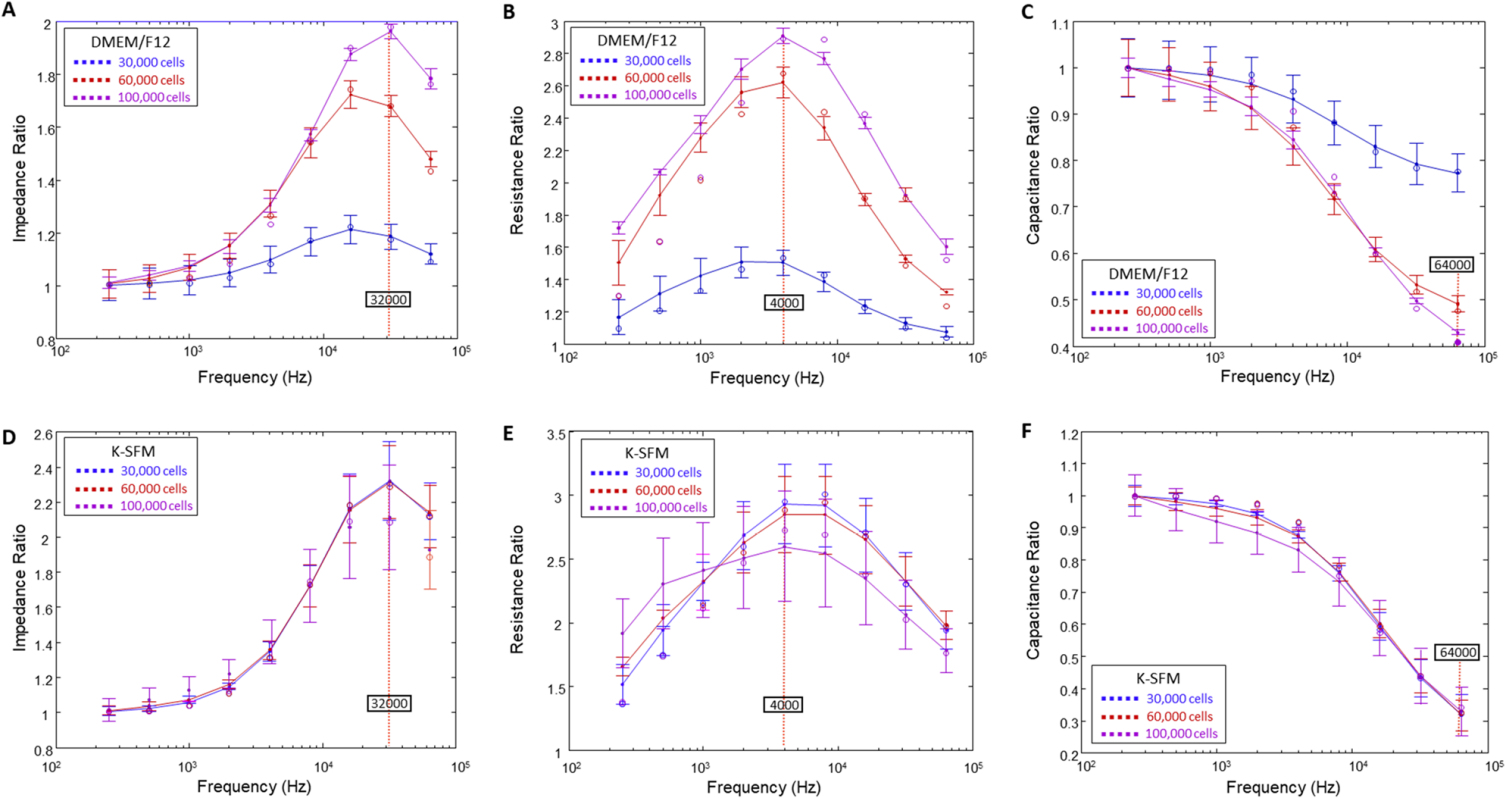
Determination of optimal AC frequencies using frequency dependence spectra. Data are presented as ratios of cell to cell-free measurements (y-axis) versus frequency (x-axis) measured at 15 h. Tracings are shown for impedance ratios with a maximum response at 32 kHz (**A**,**D**), resistance ratios with a maximum response at 4000 Hz (**B**,**E**), and capacitance ratios with a minimum response at 64 kHz (**C**,**F**) from HUCLs grown in DMEM/F12 (**A**–**C**) and K-SFM (**D**–**F**), respectively. Data shown are the mean ± SEM; n = 5/group.

Next, we aimed to dissect the influence of DMEM/F12 and K-SFM media on impedance (Z), resistance (R) and capacitance (C). When cells are challenged with an AC, pure R and C are created, which together result in the overall impedance, Z. However, to determine the optimal frequencies to use in evaluating each of these parameters, frequency dependence spectra were first measured as shown in Fig. 2. The frequency dependence of variables Z, R, and C for cells grown in DMEM/F12 at the three cell densities at T = 15 h are shown in panels A–C, respectively. Panels D–F display the same information for HUCLs grown in K-SFM at T = 15 h. At this time point, both groups have already formed confluent monolayers and are expected to have formed an intact barrier. Ratios of cell to cell-free measurements are plotted against frequency for impedance (A,D), resistance (B,E), and capacitance (C,F). As presented in Fig. 2, the impedance spectrum showed a characteristic frequency of 32 kHz, providing the greatest possible range for group comparison of cells grown in DMEM/F12 (A) and K-SFM media (D). On the other hand, we observed that 4000 Hz produces the maximum resistance in both DMEM/F12 and K-SFM media (Fig. 2B,E, respectively). Further, capacitance ratios displayed that an optimal minimal response was achieved at 64 kHz for both DMEM/F12 (Fig. 2C) and K-SFM media (Fig. 2F). However, K-SFM showed overlap between the three cell densities with greater standard deviations than DMEM/F12, thus indicating potential suboptimal conditions for HUCLs growing in the K-SFM media.

### Impedance measurements

**Figure 3:**
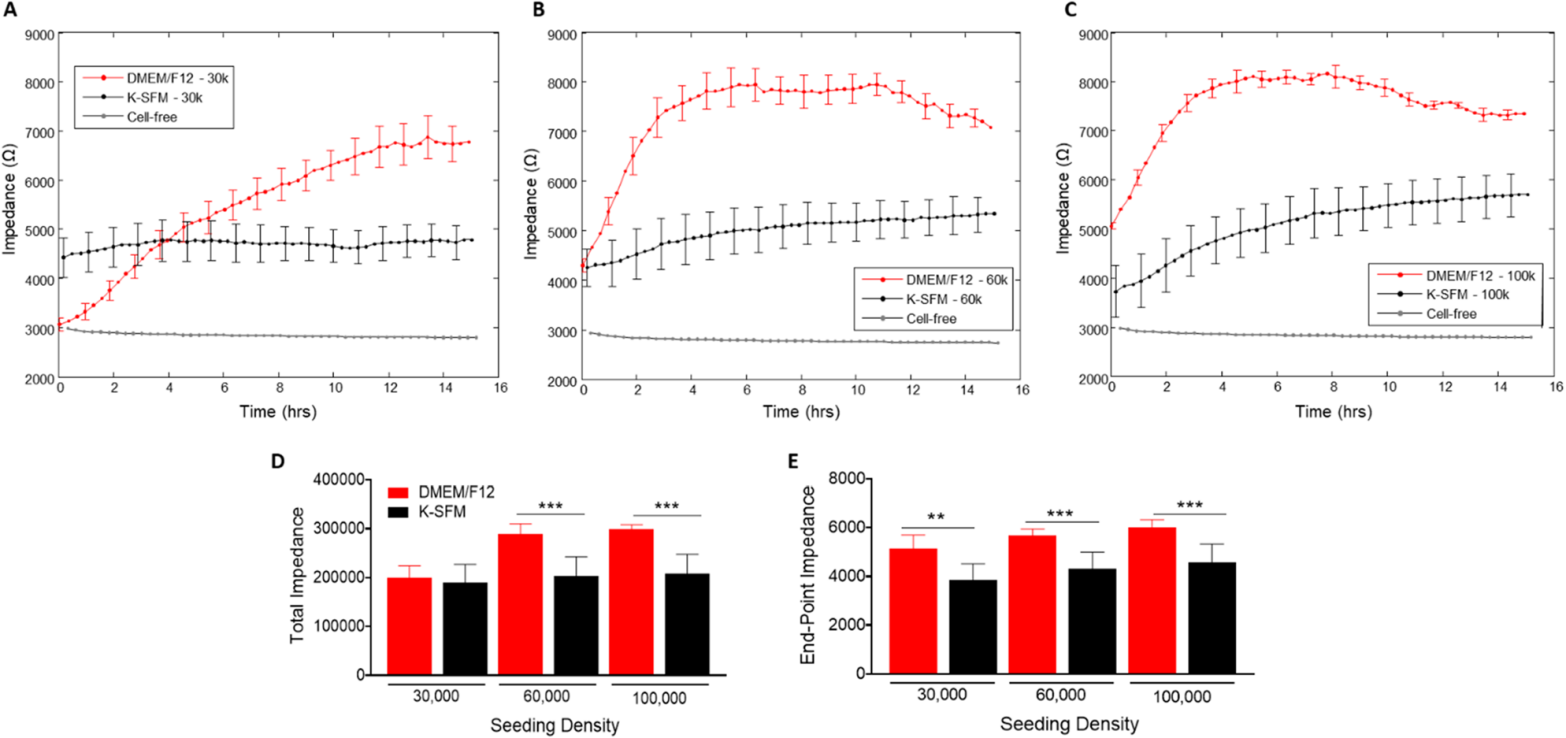
Real-time monitoring of HUCL impedance in DMEM/F12 versus K-SFM media. Impedance of HUCLs versus time, measured at an AC frequency of 32 kHz for 30,000 (**A**), 60,000 (**B**) and 100,000 (**C**) seeding densities is shown. Bar graph representation of total impedance (**D**) and end-point impedance (**E**) comparing DMEM/F12 versus K-SFM. Data shown are the mean ± SEM; n = 5/group. **p ≤ 0.01 and ***p ≤ 0.001.

Impedance (capacitive reactance) measurements, as shown in Fig. 3A–E, calculated at a high frequency of 32 kHz provide information as to when the cell monolayer is in place and confluent. This is reflected by the plateau in the impedance measurements. Cells grown in DMEM/F12 reached the plateau phase at 14–15 h for the 30,000-seeding density, and at ~ 4 h for both 60,000- and 100,000-seeding densities. Whereas HUCLs seeded at the same densities but grown in K-SFM did not display as distinct a plateau phase, indicative of poor HUCL spreading, however reached confluency at ~ 8 h for 60,000 cells and 6–8 h for 100,000 cells. This trend is further illustrated in both total (Fig. 3D) and endpoint (Fig. 3E) impedance measurements generated at 32 kHz; impedance values for HUCLs grown in DMEM/F12 were significantly higher when compared to K-SFM media. These impedance measurements indicate that the HUCLs grown in the DMEM/F12 are able to form and maintain a strong and confluent monolayer over time. Thus, indicating that investigation of HUCL barrier formation should be carried out using supplemented DMEM/F12 media in place of the classically used K-SFM media.

### Resistance measurements

**Figure 4:**
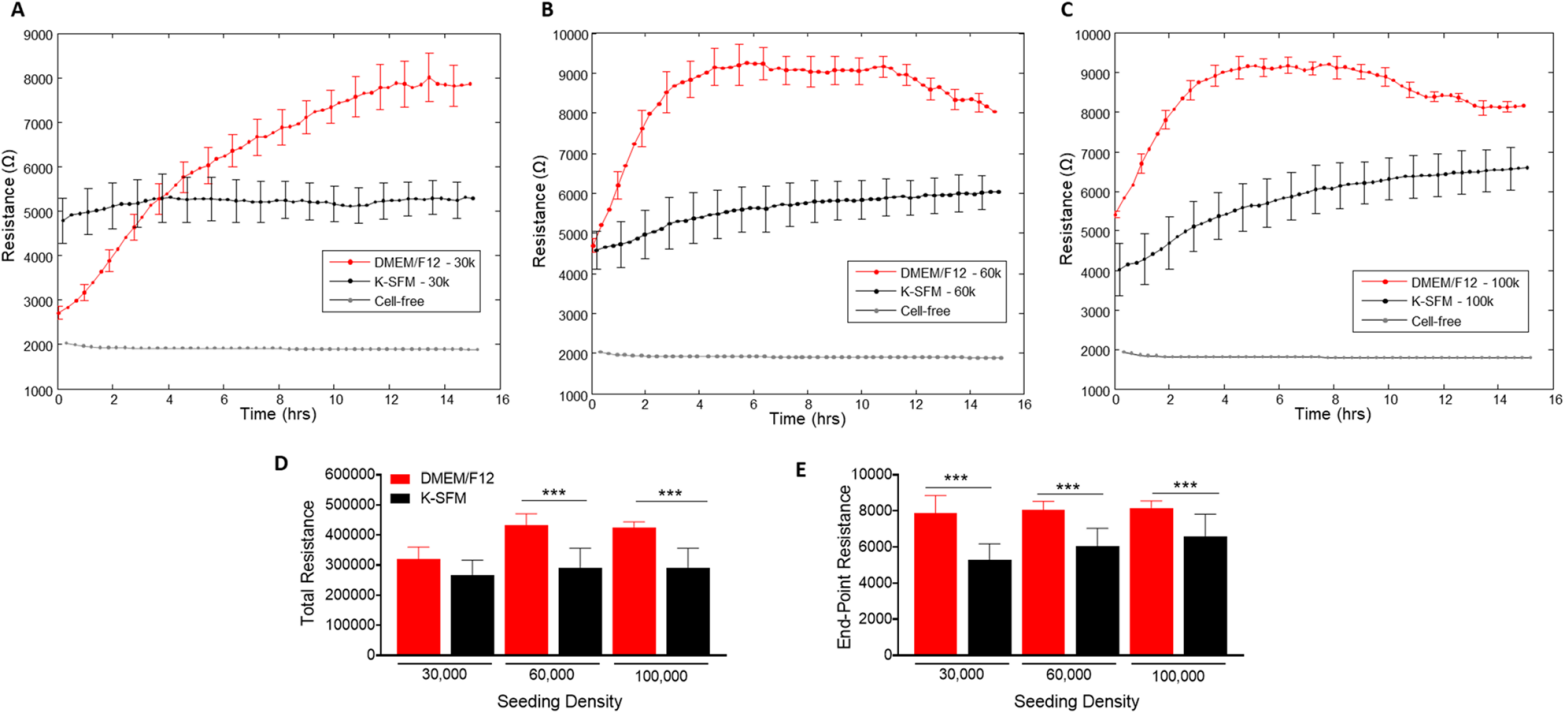
Real-time monitoring of HUCL resistance in DMEM/F12 versus K-SFM media. Resistance of HUCLs versus time, measured at an AC frequency of 4000 Hz for 30,000 (**A**), 60,000 (**B**) and 100,000 (**C**) cell seeding densities is shown. Bar graph representation of total resistance (**D**) and end-point resistance (**E**) comparing DMEM/F12 versus K-SFM. Time = 0 h denotes time of inoculation. Data shown are the mean ± SEM; n = 5/group. ***p ≤ 0.001.

Resistance measurements taken at a low frequency provides insight into the barrier formation and function. Figure 4A–C show resistance measurements generated at 4000 Hz from HUCLs seeded at the three different cell densities and grown in the two different culture media. Barrier formation is indicated by the plateau phase in each resistance profile. HUCLs grown in supplemented DMEM/F12 reached the highest resistance (9000 Ω) at 60,000 (B) and 100,000 (C) seeding densities. However, HUCLs cultured in K-SFM reached a less distinct “plateau phase” with a maximum resistance of ~ 6000 Ω. The total and endpoint resistance values shown in Fig. 4D and E, respectively, were generated out to a maximum of 16 h as determined by the barrier formation plateaus for both groups of media. Total resistance values were significantly higher in DMEM/F12 versus K-SFM at seeding densities of 60,000 and 100,000 cells. Furthermore, endpoint resistance measurements showed that all three cell densities grown in DMEM/F12 were significantly higher compared to K-SFM cells, indicating the formation of tighter and stronger epithelial cell barriers when grown in DMEM/F12. Collectively, these results support that the optimal growth, barrier formation, and sustaining conditions for HUCLs are best carried out in the DMEM/F12 media. Without the supplementation, as indicated by HUCLs grown in K-SFM, “mature” barriers are not as well formed, thus providing further evidence for the use of supplemented DMEM/F12 media when studying corneal epithelial function in vitro. These trends were observed when carried out to later time points, as well, as shown at 70 h in Supplemental Fig. 1.

### Capacitance measurements

**Figure 5:**
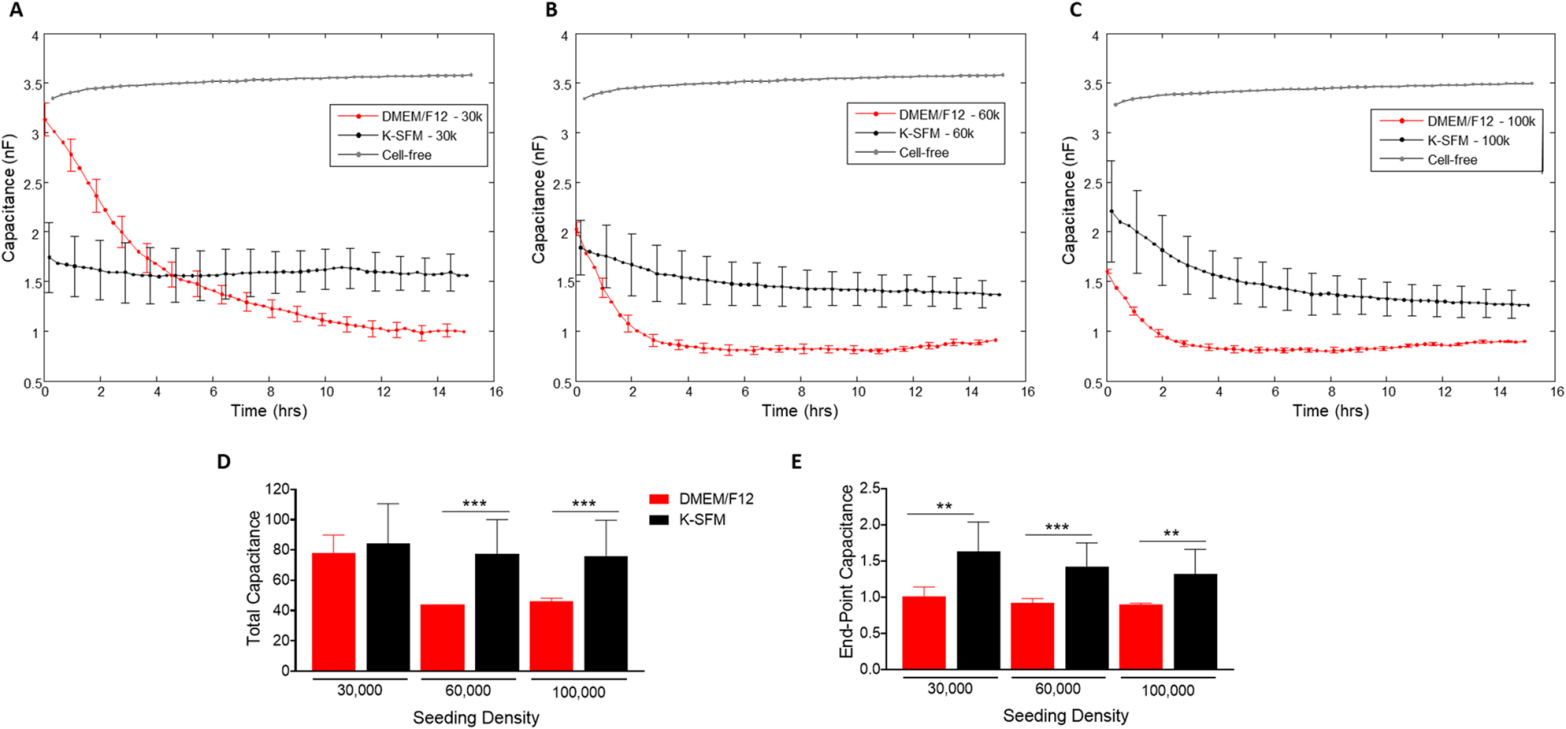
Real-time monitoring of HUCL capacitance in DMEM/F12 versus K-SFM media. Capacitance of HUCLs versus time, measured at an AC frequency of 64 kHz is shown for 30,000 (**A**), 60,000 (**B**), and 100,000 (**C**) cell seeding density. Total capacitance (**D**) and end-point capacitance (**E**) comparing DMEM/F12 versus K-SFM are represented by bar graphs. Data shown are the mean ± SEM; n = 5/group. **p ≤ 0.01 and ***p ≤ 0.001.

As with the resistance measurements described above, the growth characteristics of HUCLs were observed in the real-time formation of confluent cell layers and measured as capacitance (Fig. 5A–E). Cells grown in the supplemented DMEM/F12 media displayed more efficient cell spreading at all seeding densities compared to cells grown in K-SFM. At the 30,000 seeding cell density (A), cells grown in DMEM/F12 reached a confluent monolayer between 8 and 10 h. HUCLs seeded at 60,000 and 100,000 cells grown in the DMEM/F12 formed a confluent monolayer between 2 and 4 h (B and C). Whereas HUCLs at either 30,000 or 60,000 seeding densities grown in K-SFM exhibited much less efficient cell spreading. Cells grown in K-SFM were able to establish a confluent layer; however, it took longer at 5–6 h as reflected by capacitance. To further illustrate the differences between DMEM/F12 and K-SFM in the formation of a confluent layer, total and endpoint capacitance measurements are also shown. Total capacitance (Fig. 5D) was significantly lower at 60,000 and 100,000 cell seeding densities for DMEM/F12 compared to K-SFM. As shown in Fig. 5E, endpoint capacitance for all three seeding densities was significantly decreased in DMEM/F12, as well. Because of the inverse relationship between capacitance and cell spreading, it is indicated that the DMEM/F12 media better supports the growth, spreading and formation of a confluent cellular layer compared to the classically used K-SFM growing conditions.

### Mathematical modeling of the R data—R_b_, α and C_m_

**Figure 6:**
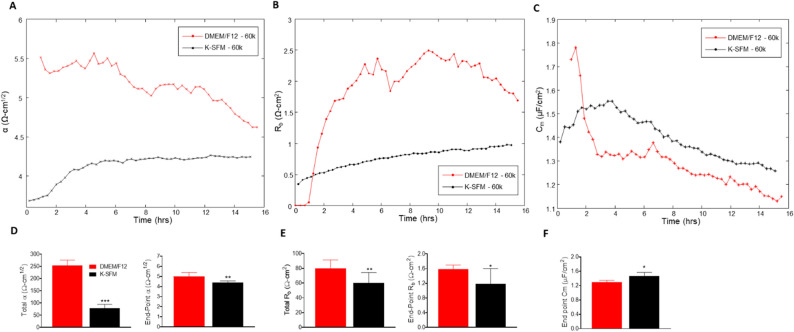
Mathematical modeling of α, R_b_, and C_m_ for HUCLs grown in DMEM/F12 versus K-SFM media. Modeled parameters, α (**A**), R_b_ (**B**), and C_m_ (**C**) were traced over 15 h for cells seeded at 60,000. Time = 0 denotes time of inoculation. Bar graphs represent total and end-point values from DMEM/F12 versus K-SFM media for α (**D**) and R_b_ (**E**); end-point only is shown for C_m_ (**F**). Data shown are the mean ± SEM; n = 5/group. *p ≤ 0.05, **p ≤ 0.01 and ***p ≤ 0.001.

The ECIS software has the ability to model the impedance into parameters that distinguish between cell–cell (R_b_) and cell–matrix (α) adhesions, as well as membrane capacitance (C_m_). R_b_ is the resistivity of cell–cell contacts to the current flow. α measures the impedance contributions arising from the cell–electrode junctions. Therefore, the contribution of R_b_, α, and C_m_ to the observed changes in previous experiments was calculated by fitting a mathematical model developed by Giaever and Keese^8^. R_b_, α, and C_m_ values were determined from HUCLs at the 60,000-cell seeding density grown in DMEM/F12 compared to K-SFM media and are presented in Fig. 6A–F.

The constructed parameter α, indicating the strength of interaction between the cells with the basal substrate, is higher in cells grown in DMEM/F12 compared to K-SFM throughout the entire time course (Fig. 6A). These results combined with total and endpoint α measurements (Fig. 6D), which are also significantly higher for HUCLs grown in DMEM/F12 compared to K-SFM, indicate that cells grown in the DMEM/F12 media create stronger cellular attachments to the basal substrate. These data may also contribute to the overall differences seen in the resistance values between HUCLs grown in DMEM/F12 versus K-SFM.

Furthermore, R_b_ values, which reflect paracellular barrier strength, were higher in HUCLs cultured in DMEM/F12 media when compared to HUCLs grown in K-SFM media (Fig. 6B). This observed increase in barrier function is further demonstrated by corresponding total and endpoint R_b_ values (Fig. 6E), where HUCLs grown in DMEM/F12 displayed significantly higher R_b_ values than K-SFM media. In addition to the α value, the fact that HUCLs grown in DMEM/F12 displayed higher R_b_ values compared to the cells grown in K-SFM indicates that stronger cell–cell interactions are also playing an underlying role in the overall differences observed in resistance.

C_m_ or the capacitance of the cell membrane, shown in Fig. 6C, is indicative of temporal alterations in membrane thickness and composition. Additionally, C_m_ measurements are used to determine if variations in capacitance are only due to changes in electrode coverage or are a function of microvariations in the apical membrane structures. Total C_m_ is not presented since confluent monolayers are required to model this parameter, which did not consistently occur at earlier timepoints (< 6 h) for cells grown in K-SFM. As a result, only end-point C_m_ is shown, which is significantly lower in HUCLs grown in DMEM/F12 compared to K-SFM (Fig. 6F). Therefore, the interpretation from the data is that the differences in C_m_ are due to differences in electrode coverage and not membrane structure.

### Cellular morphology

**Figure 7:**
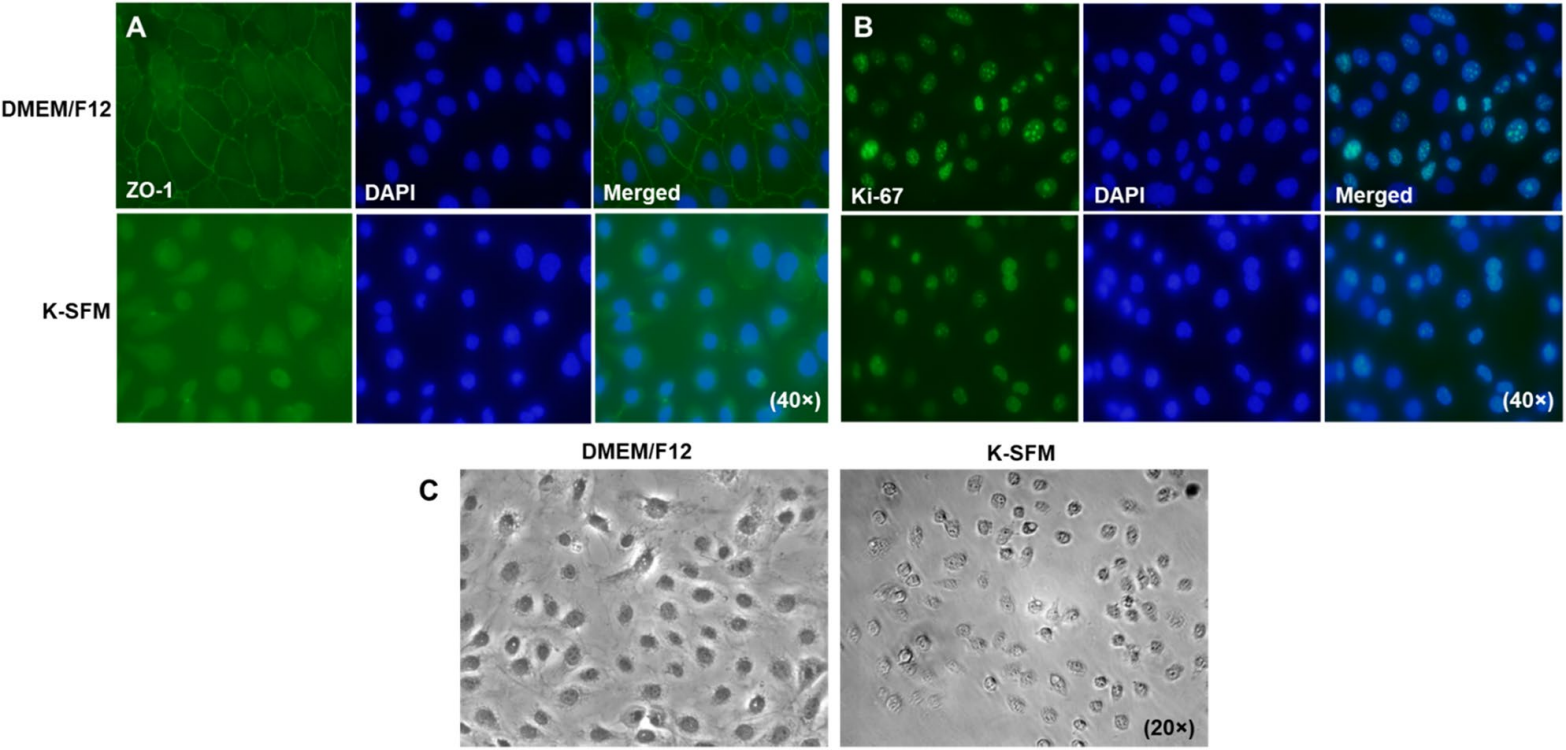
Visual observations of HUCLs cultured in DMEM/F12 and K-SFM. Levels of ZO-1 (**A**) and Ki-67 (**B**) were assessed by immunofluorescence after 5 days of culturing in either DMEM/F12 or K-SFM media. ZO-1 and Ki-67 are shown in green with nuclei of cells counterstained with DAPI shown in blue. Images are shown at 40 × . Phase-contrast microscopy (**C**) images of HUCLs grown in both DMEM/F12 and K-SFM are shown at 20 × .

To correlate our functional studies with morphological differences, levels of ZO-1, a component of the tight junction complex required for paracellular signaling, were determined qualitatively by immunostaining (Fig. 7A). HUCLs grown in DMEM/F12 exhibit a continuous linear pattern of ZO-1 staining along cell–cell borders with DAPI stained nuclei. This characteristic cobblestone like pattern was not observed in cells grown in K-SFM. In fact, cells appeared more rounded with fewer cell–cell interactions and very little positive ZO-1 staining. HUCLs were also stained for the proliferation marker, Ki-67, as shown in Fig. 7B. Both groups showed similar staining patterns indicating similar proliferation rates between the two types of media. However, phase-contrast microscopy (Fig. 7C) reiterated what was observed with the ZO-1 staining. HUCLs grown in DMEM/F12 display a polygonal shape that are tightly joined with little intercellular space. Despite similar cell numbers, those grown in K-SFM did not appear squamous or cuboidal, but instead had a round appearance with very few cell–cell contacts.

### Response to wounding stimulus

**Figure 8:**
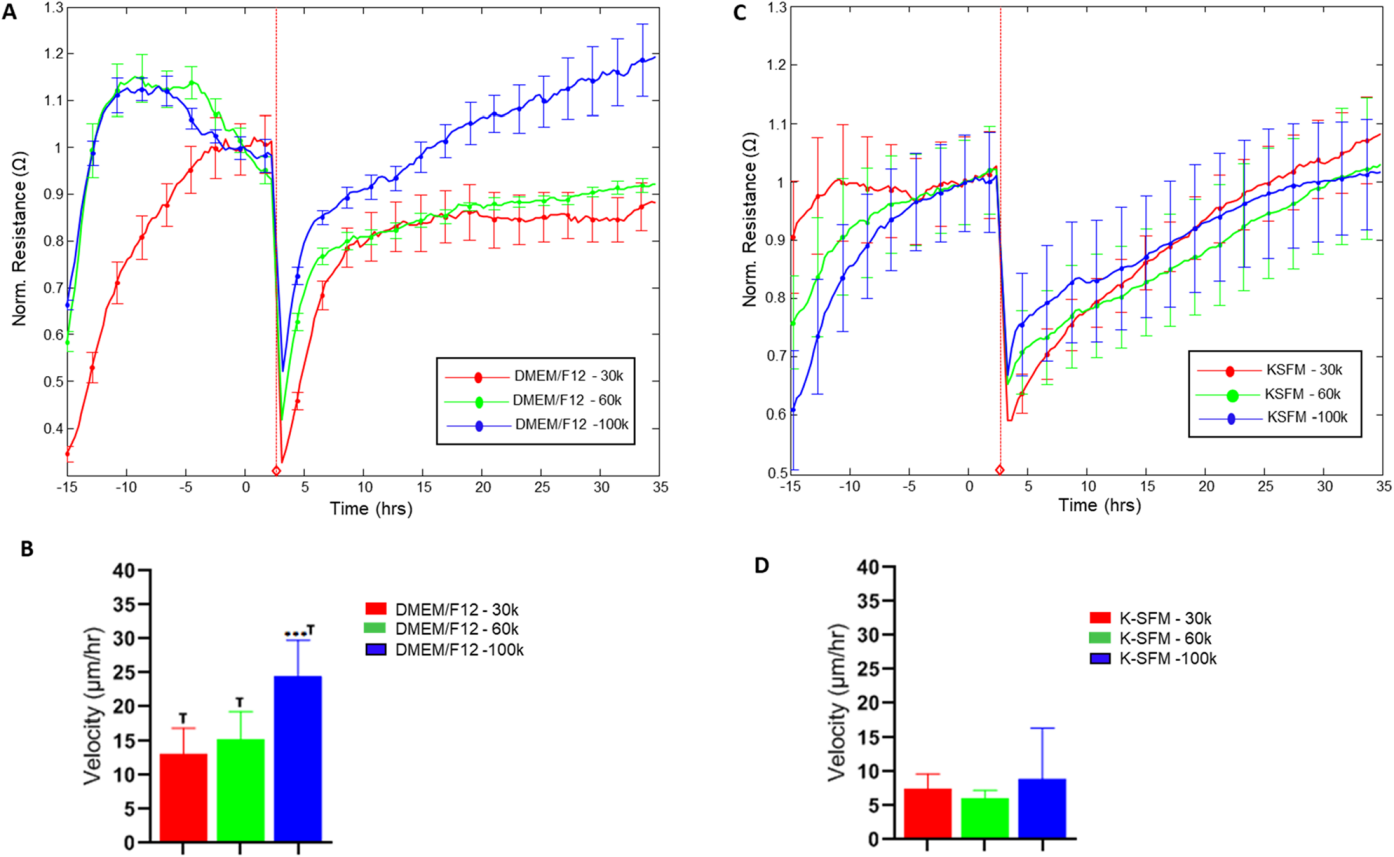
Wound healing response of HUCLs grown in DMEM/F12 versus K-SFM media. Normalized resistance of HUCLs versus time, measured at an AC frequency of 4000 Hz for 30,000, 60,000 and 100,000 cell seeding densities is shown for DMEM/F12 (**A**) and K-SFM (**B**). Bar graph representation of cell velocity of migrating cells (**C**,**d**) for both groups over time. Time = 0 h denotes time of wounding. Data shown are the mean ± SEM; n = 5/group. ***p ≤ 0.001; † *p* ≤ 0.01 between DMEM/F12 and K-SFM groups.

To investigate further into the functional impact of the observed differences in culture conditions, HUCLs were preliminarily assessed for potential differences in response to wounding, as shown in Fig. 8. Rates of recovery following wounding were determined as velocity of cell migration (derived from normalized resistance values) for cells cultured in DMEM/F12 (A, B) and K-SFM (C, D) for each seeding density over time until 100% of the normalized resistance prior to wounding was reached. Differences in recovery time were observed between the two media for all seeding densities with cell velocities approximately 50% decreased in K-SFM compared to DMEM/F12. This differential response to a common stimulus such as wounding suggests that culturing conditions have an impact that extends beyond the initial culturing response by HUCLs.

## Discussion

The ECIS biosensor technology is a powerful tool to measure and model key aspects of cellular function that provide insight into changes in cellular structure. The current study highlights the comprehensive analyses generated by ECIS for in vitro study of human corneal epithelial cells. These quantitative assessments directly relate to cellular function, particularly spreading and barrier formation, which are key to maintaining corneal homeostasis as well as contributing factors to disease pathogenesis. Taken together, real-time measurements of parameters that include impedance (Z), resistance (R), capacitance (C), R_b_, α, and C_m_ allow for a more extensive understanding regarding the structural and functional aspects of a cell under in vitro conditions than more traditional techniques that are standardly used.

Real-time impedance (Z) measurements are indicative of cellular motility and the rate at which the corneal epithelial barrier is formed. Impedance is then broken down into resistance (R) and capacitance (C) to allow for differentiation of adhesion, spreading, and proliferation—a major limitation to traditional in vitro approaches^9^. Resistance is the part of impedance that best defines barrier quality and function because it does not consider capacitive components from the membrane, electrode or cell medium^10^. Resistance itself is directly determined by the cell through cell–cell and cell-substrate interactions that block the flow of current. When cells attach to the substrate, for example, the flow of current will become increasingly restricted as the cells spread over the electrode. As a result, at higher frequencies, the capacitance decreases in a linear correlation to the percentage of open electrode area^9^. In this case, adhesion, spreading and proliferation are quantified at a frequency higher than 40 kHz. Complete cellular coverage of the electrode is indicated by the flattening out of the slope related to the capacitance curve. It is important to underscore that capacitance measurements indicate coverage, but resistance data reveal when a mature barrier is formed. To this end, we show that HUCLs grown in DMEM/F12 generated a confluent monolayer between 2 and 3 h (indicated by capacitance), but a functional barrier was not established until 4–6 h (indicated by resistance). This highlights the power of ECIS in allowing for a more accurate interpretation of cellular changes, while also calling attention to the importance of precise data interpretation.

Impedance measurements acquired across several frequencies can be mathematically modeled using the ECIS software to obtain important parameters that together relate to the overall barrier formation of these cells. These values are R_b_ (resistance of the paracellular barrier), α (resistance of the cell:substrate barrier) and C_m_ (capacitance of the cell membrane). ECIS is the only impedance measuring system to use multifrequency AC currents, which results in more robust data compared to systems like xCELLigence by ACEA, which only generates data from a single AC frequency^11^. Separating cell–cell (R_b_) adhesions from cell-substrate (α) interactions provide key insights into understanding which elements of the corneal epithelial barrier are contributing to the overall barrier strength and resistance. R_b_ is the resistivity of cell–cell contacts to the current of flow. As a result, high R_b_ implies a low permeability toward the current flow and thus stronger cell–cell adhesions. Alpha (α) is a measure for the impedance contributions that arise from the cell-substrate junctions. The model to quantify cell–cell and cell-substrate contacts was introduced in 1991 by Giaever and Keese and assumes that cells are circular, disc-shaped objects that have an insulating membrane, hover over the electrode, and are filled with a conducting electrolyte^8^.

This initial study first aimed to determine the experimental parameters (media type, supplementation details, seeding densities, and frequency range) that would allow for optimal in vitro assessment of corneal epithelial cell function using the ECIS system. Previous in vitro studies investigating corneal epithelial cells have used serum-containing media and others have used serum-free media. This likely due to the possibility that serum may influence the proliferation and differentiation of corneal epithelial cells^12^ since it may contain growth factors that unknowingly inhibit or activate cellular growth. Our ECIS studies were initially carried out using the traditional corneal epithelial cell media, K-SFM for the reason stated above. K-SFM is a serum-free medium with L-glutamine and supplemented with recombinant epidermal growth factor and bovine pituitary extract. However, extreme variations in the data were consistently observed and surprisingly weak readings for impedance, resistance and capacitance were generated for HUCLs compared to other studies looking at epithelial cell barrier formation. We had previously published on ECIS assessment of the human retinal pigment epithelial cell line, ARPE-19, which is widely used as an alternative for primary retinal pigment epithelial cells^13^. This cell line is grown in DMEM/F12 medium as a standard^13,14^. DMEM is a basal medium that contains increased amino acid and vitamin concentrations. F12 additionally provides biotin, putrescine, lipoic acid, proline, copper, and zinc. The literature further revealed studies of different types of epithelial cells (retinal epithelial cells^13^ and kidney epithelial cells^15^) also utilized DMEM/F12 media supplemented with 10% FBS. Therefore, we compared both K-SFM media with no FBS supplementation and supplemented DMEM/F12.

HUCLs maintained in DMEM/F12 media were found to outperform cells similarly grown in K-SFM across all parameters. This was evidenced by real-time impedance (Z) measurements indicating faster barrier formation and better motility. Not only did these cells migrate more quickly but they also formed a stronger corneal epithelial barrier than cells grown in K-SFM media. Resistance measurements taken from DMEM/F12 were almost 2 × greater than that observed in K-SFM. In addition, a corneal epithelial barrier was established in ~ 1/3 of the time it took for cells grown in K-SFM, as indicated by the R plateau. Capacitance data revealed correlative findings where cells grown in DMEM/F12 formed a monolayer in 2–3 h compared to ~ 6 h for cells grown in K-SFM. Furthermore, the slope of the capacitance curve is much steeper for HUCLs grown in DMEM/F12 compared to K-SFM, reflecting cells that are much more motile and spread more quickly across the area of the electrode. Not surprisingly, HUCLs grown in DMEM/F12 also had stronger cell–cell (R_b_) and cell-substrate (α) interactions than cells grown in K-SFM. These data reveal that the differences in R_b_ and α are the underlying reasons for such drastic differences in the resistance and capacitance readings between the two groups of media. HUCLs grown in DMEM/F12 are able to form stronger tight junctions between neighboring cells and also create stronger interactions with the basal substrate. This was further supported by substantially reduced ZO-1 staining and more intercellular space observed with K-SFM media. Thus, it appears that DMEM/F12 provides essential nutrients needed to migrate efficiently and form a strong and tight corneal epithelial cell barrier under in vitro conditions. This is significant because previously, K-SFM was thought to be the ideal culture medium standard for corneal epithelial cells. However, as observed using the ECIS system, these cells demonstrate poor cell migration and reduced barrier formation in K-SFM media.

As shown, 60,000 cells per well generated the optimal data for HUCLs. Seeding densities > 100,000 cells were also evaluated and are included as Supplemental Fig. 2, which resulted in barrier function stress and greater standard deviations for impedance, resistance, and capacitance parameters. This work also highlights the sensitivity of the ECIS technology and the importance of optimizing for cell density.

In conclusion, our study utilizes ECIS to monitor cellular behavior in HUCLs, while optimizing cell culturing conditions in vitro. Without a comprehensive assessment of cellular behavior in real-time, it is likely that traditional in vitro conditions may miss the mark regarding functional assessments. In fact, phase-contrast microscopy imaging of cells seeded at a density of 60,000/well demonstrate that both DMEM/F12 and K-SFM appear confluent by 8 h (Supplemental Fig. 3) despite the clear functional differences reported herein. It is also noted that the morphology of cells grown in K-SFM appears more spindle-like as opposed to rounded cells grown in DMEM/F12. Further, K-SFM cells do not form the expected groupings observed in DMEM/F12, consistent with the lack of cell:cell adhesions and reduced barrier function as determined by ECIS. The current study not only highlights the importance of optimization but also data interpretation. For example, assuming that a confluent monolayer equates to an optimally functional barrier regarding the latter point. This is further demonstrated by the preliminary wounding data, which revealed that cell migration was markedly influenced by culturing conditions. These results suggest that the composition of culture media plays a consequential role in in vitro functional assessments. In future studies, ECIS will be utilized to evaluate the functional influence of potential therapies being developed for microbial keratitis or dry eye disease on epithelial wound healing migration and subsequent re-establishment of a mature epithelial barrier. Collectively, the ECIS system provides an invaluable opportunity to elucidate cellular migration and barrier formation in vitro.

## Materials and methods

### Human telomerase-immortalized corneal epithelial cell (HUCL) culture

HUCLs, kindly provided by Dr. Fu-Shin Yu’s laboratory, were used in these studies. These cells, infected with a retroviral vector encoding human telomerase reverse transcriptase to create an immortalized cell line, have been previously described and appropriately confirmed as an applicable in vitro model for corneal epithelial cell investigation^16^. In the current study, HUCLs were maintained in two different media for all experiments: Dulbecco’s modified Eagle’s medium–nutrient mixture F12 (DMEM/F-12; Thermo Scientific, Wyman, MA, USA) supplemented with 10% fetal bovine serum (FBS; Atlantic Biological, Norcross, GA, USA) and 1% penicillin/streptomycin (PS) and keratinocyte-serum-free medium (K-SFM) supplemented with growth factors (EGF and bovine pituitary extract; Invitrogen-Life Technologies, Carlsbad, CA, USA). Cells were used between passages 3–5 for all experiments and maintained in a humidified 5% CO_2_ incubator at 37 °C.

### Conducting ECIS experiments and modeling

HUCL assessments were determined by observing changes in transcellular electrical resistance (TER) using the ECIS Zϴ system (Applied Biophysics Inc, Troy, NY, USA)^13^. Electrode arrays (96W1E+) (Applied Biophysics Inc., Troy, NY, USA) are 96-well plates with each well containing two circular 350 µm diameter active electrodes. The total area of the electrode is 0.256 mm^2^. Arrays were pretreated with 100 µL of 100 µM cysteine for 30 min, followed by coating with fibronectin collagen (FNC Coating Mix; Athena Environmental Service, Inc., Baltimore, MD, USA) for 2 min. Prior to seeding the wells, electrode impedance values were stabilized, as recommended by the manufacturer, to minimize electrode drift during the experiment. Wells were then inoculated with HUCLs at five different seeding densities: 30,000, 60,000, 100,000, 200,000 and 500,000 cells per well in 200 µL of media (n = 5 wells seeded for each group). The plate was maintained for ~ 15 h at a constant current of approximately 1 µA to each well. At 17 h, automated wounding was induced using the following AC parameters: wound time − 30 s, current − 1500 µA, frequency − 60 kHz to effectively, yet precisely, kill cells over each electrode and produce two wounded areas in the cell monolayer. The run was carried out under multiple frequencies ranging from 1000 to 64 kHz and continuously monitored with measurements taken roughly every 2 min. Impedance values were normalized to the impedance values generated by cell-free electrodes. ECIS measurements were acquired from five replicates per experiment.

### Data analysis and modeling

ECIS measurements were acquired for overall resistance (R), impedance (Z), and capacitance (C) at 4 kHz, 32 kHz and 64 kHz, respectively, as a function of time. Parameters were determined by comparing cell data to cell-free electrodes, per the manufacturer’s recommendation. Multi-frequency scans were used to measure impedance also as a function of frequency and represented as a three-dimensional plot with frequency along the x-axis and time along the z-axis.

The ECIS technology is enhanced by the ability to apply mathematical modeling to derive three parameters that reflect the properties of cells: R_b_ (the electrical resistance between cells, Ω cm^2^), α (the basolateral resistance between the HUCLs and substrate, Ω cm^1/2^), and C_m_ (the capacitance of the HUCL cell membrane, µF/cm^2^). The ECIS software was also used to model these parameters as total and end point values as previously described^2^. The parameter R_b_ is crucial to modeling in vitro epithelial barrier function, as it describes the tightness of the intercellular space, which is highly dependent on cell–cell junctions. The two remaining parameters, α and C_m_, are indicative of the current flow below or through cells, respectively. ECIS biosensor technology is the only technology currently available that can model each of these important cellular parameters. However, average capacitance of cell membranes cannot distinguish between apical and basal membranes. Drift Correction and Model Fit RMSE (root mean square error) values were used to validate the modeled data. The migration velocity was calculated by dividing the total distance that HUCLs migrated over the electrode radius (175 µm) by the total time it took to recover 100% of normalized resistance.

### Immunofluorescence of ZO-1 and Ki-67

As a complementary approach to ECIS, epithelial barrier integrity was assessed by staining for the one of the epithelial tight-junction proteins, ZO-1. The proliferation maker, Ki-67, was also used to determine any differences in cellular morphology. Briefly, HUCLs were seeded in a 6-well plate containing FNC-coated coverslips at a density of 300,000 cells per well and cultured for 5 days (37 °C, 5% CO_2_). On day 5 cells were visually confirmed as confluent, coverslips were washed with PBS and fixed by Z-Fix (Anatech Ltd, Battle Creek, MI, USA), a 10% aqueous buffered zinc formalin, for 15 min at room temperature. Cells were then permeabilized in 0.05% triton X-100 for 15 min on ice. Next, the coverslips were washed 2 × with PBS, followed by blocking in 5% BSA for 1 h. Cells were then incubated overnight at 4 °C with a ZO-1 mAb (1:100) (D6L1E-Alexa Fluor 488, Cell Signaling Technologies, Danvers, MA, USA) or Ki-67 mAb (1:100) (FITC mouse anti-Ki-67 set, BD Biosciences, San Diego, CA, USA). Coverslips were washed 2 × with PBS and mounted using Prolong Diamond plus DAPI (Invitrogen, Eugene, OR, USA). Images, including both fluorescent and phase-contrast, were taken with an Olympus BX53 microscope.

### Statistical analysis

Statistical analyses were performed using GraphPad Prism 7.03. Data are presented as mean ± SEM for one representative experiment. Differences between multiple groups were determined by a two-way ANOVA with multiple comparisons (main mean effect). Comparisons made between two groups were made using an unpaired, Student’s t-test. Significance was determined by *p*-values < 0.05 and represented as follows: *p < 0.05, **p < 0.01, ***p < 0.001, unless indicated otherwise.

## Supplementary Information

Supplementary Information.

## Data Availability

All data generated during and/or analysed during the current study are included in this published article.

## References

[1] Reiss B, Wegener J (2015). Impedance analysis of different cell monolayers grown on gold-film electrodes. Annu. Int. Conf. IEEE Eng. Med. Biol. Soc..

[2] Robilliard LD (2018). The importance of multifrequency impedance sensing of endothelial barrier formation using ECIS technology for the generation of a strong and durable paracellular barrier. Biosensors (Basel).

[3] Stolwijk JA, Matrougui K, Renken CW, Trebak M (2015). Impedance analysis of GPCR-mediated changes in endothelial barrier function: Overview and fundamental considerations for stable and reproducible measurements. Pflugers Arch..

[4] Cavet ME (2010). Effect of a novel multipurpose contact lens solution on human corneal epithelial barrier function. Cont. Lens Anterior Eye.

[5] Stepp MA, Menko AS (2021). Immune responses to injury and their links to eye disease. Transl. Res..

[6] Li H (2006). Herpes simplex virus 1 infection induces the expression of proinflammatory cytokines, interferons and TLR7 in human corneal epithelial cells. Immunology.

[7] Sridhar MS (2018). Anatomy of cornea and ocular surface. Indian J. Ophthalmol..

[8] Giaever I, Keese CR (1991). Micromotion of mammalian cells measured electrically. Proc. Natl. Acad. Sci. U.S.A.

[9] Wegener J, Keese CR, Giaever I (2000). Electric cell-substrate impedance sensing (ECIS) as a noninvasive means to monitor the kinetics of cell spreading to artificial surfaces. Exp. Cell Res..

[10] Szulcek R, Bogaard HJ, van Nieuw Amerongen GP (2014). Electric cell-substrate impedance sensing for the quantification of endothelial proliferation, barrier function, and motility. J. Vis. Exp..

[11] Kho D (2015). Application of xCELLigence RTCA biosensor technology for revealing the profile and window of drug responsiveness in real time. Biosensors (Basel).

[12] Kruse FE, Tseng SC (1993). Serum differentially modulates the clonal growth and differentiation of cultured limbal and corneal epithelium. Invest. Ophthalmol. Vis. Sci..

[13] Guerra MH (2021). Real-time monitoring the effect of cytopathic hypoxia on retinal pigment epithelial barrier functionality using electric cell-substrate impedance sensing (ECIS) biosensor technology. Int. J. Mol. Sci..

[14] Ahmado A (2011). Induction of differentiation by pyruvate and DMEM in the human retinal pigment epithelium cell line ARPE-19. Invest. Ophthalmol. Vis. Sci..

[15] Lo CM, Keese CR, Giaever I (1995). Impedance analysis of MDCK cells measured by electric cell-substrate impedance sensing. Biophys. J..

[16] Zhang J (2004). Role of EGFR transactivation in preventing apoptosis in Pseudomonas aeruginosa-infected human corneal epithelial cells. Invest. Ophthalmol. Vis. Sci..

