# Supplementary material for: Functional optimization of electric cell-substrate impedance sensing (ECIS) using human corneal epithelial cells
Source: Sci Rep. 2022 Aug 19;12:14126. doi: 10.1038/s41598-022-18182-z (PMC9391335; doi:10.1038/s41598-022-18182-z)
Supplement: Supplementary file 1 — Supplementary Information. [file 41598_2022_18182_MOESM1_ESM.pdf]

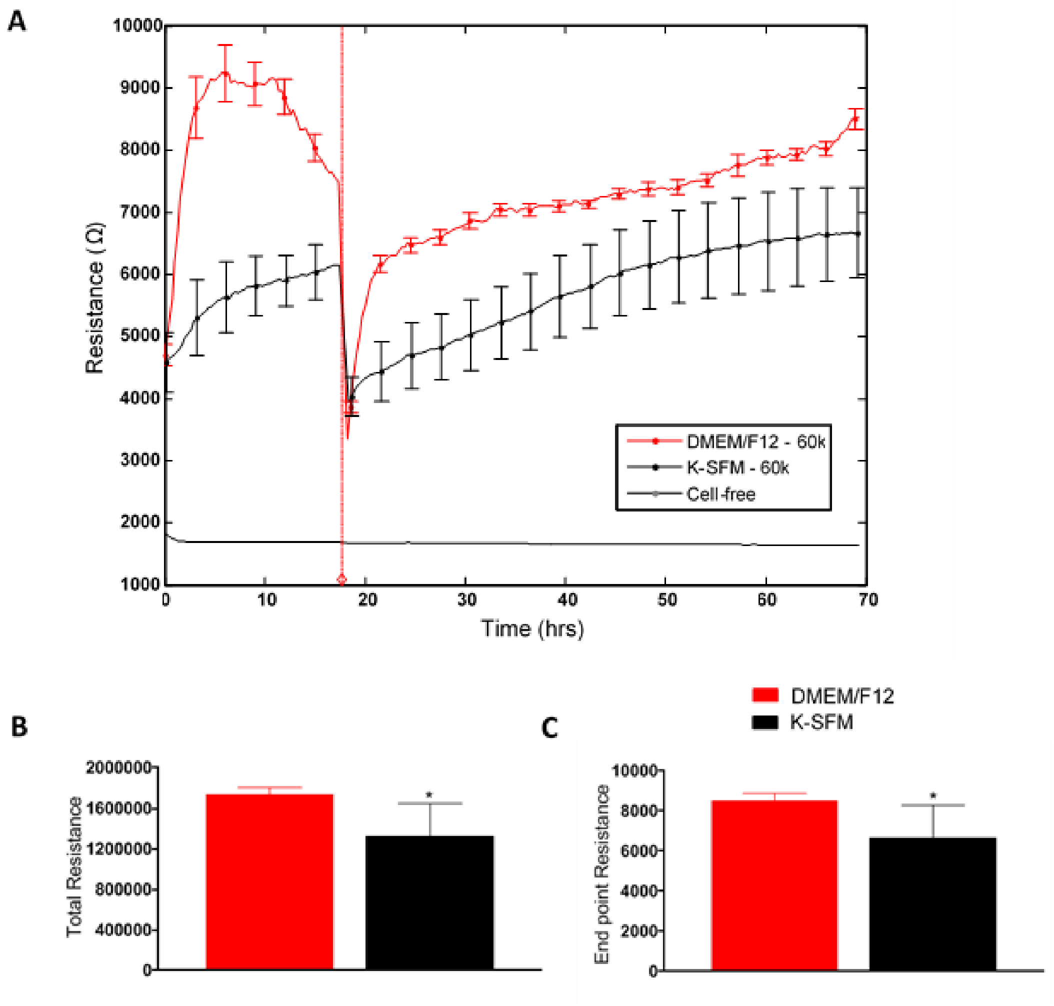

Supplemental Figure 1. Real-time monitoring of HUCL resistance in DMEM/F12 versus K-SFM over time. Resistance of HUCLs versus time, measured at an AC frequency of 4000 Hz for 60,000 cells (***A***). Bar graph representation of total resistance (***B***) and end-point resistance (***C***) comparing DMEM/F12 versus K-SFM out to 70 hours. Vertical red line denotes wounding event at T = 18 hours. Data shown are the mean $\pm$ SEM; n = 5/group. * $p \leq 0.05$.

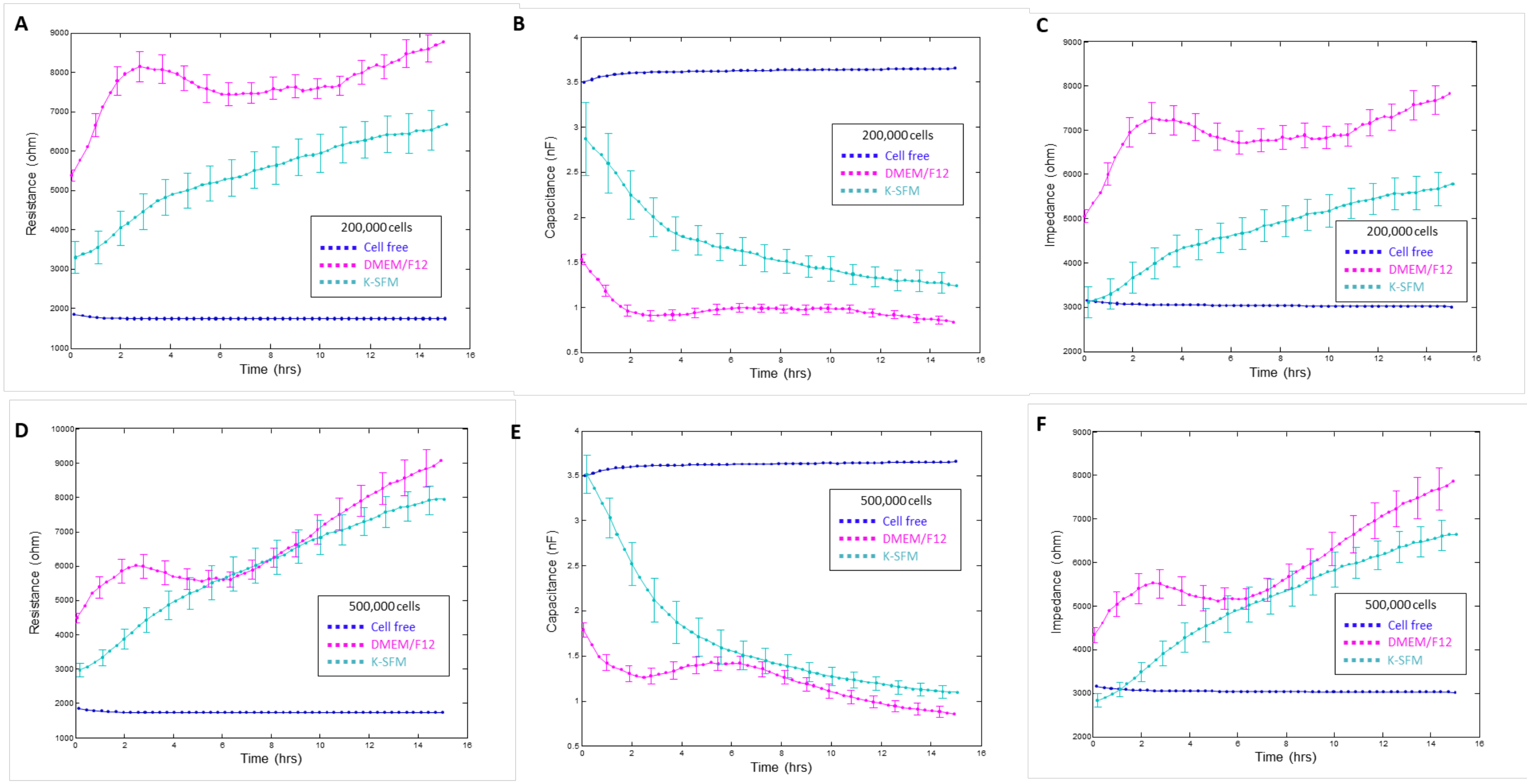

Supplemental Figure 2. Real-time monitoring of HUCLs seeded at 200,000 (***A - C***) and 500,000 (***D - F***) cells per well in DMEM/F12 versus K-SFM. Tracings are shown for resistance measured at 4000 Hz (***A, D***), capacitance measured at 64 kHz (***B, E***), and impedance measured at 32 kHz (***C, F***). Data shown are the mean ± SEM; n = 5/group.

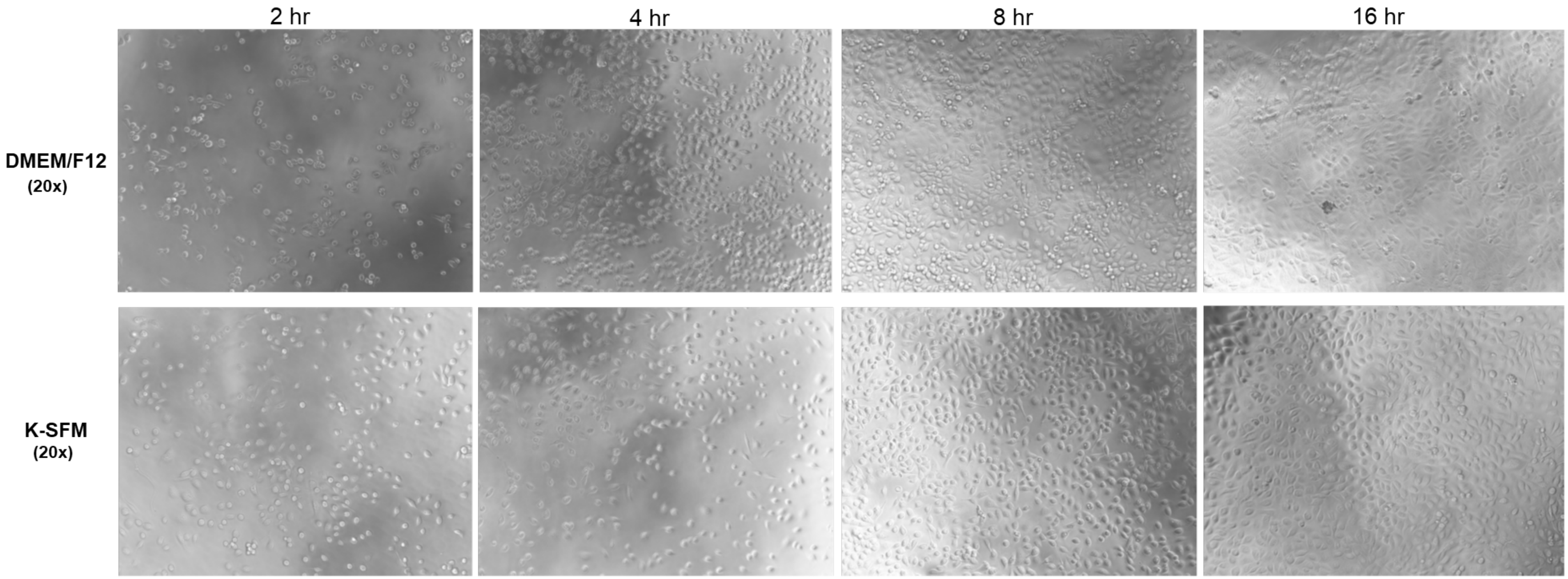

Supplemental Figure 3. Phase-contrast microscopy images of HUCLs seeded at 60,000 cells per well in DMEM/F12 versus K-SFM using a 96-well plate. Representative images are shown to demonstrate confluency of cells between the two groups of media at 2 hr, 4 hr, 8 hr, and 16 hr after initial seeding. Images were captured using EVOS FL Auto Microscope (Life Technologies) at 20× magnification; n = 6/group.
